# Supplementary material for: Antimicrobial Resistance Genes Analysis of Publicly Available Staphylococcus aureus Genomes
Source: Antibiotics (Basel). 2022 Nov 16;11(11):1632. doi: 10.3390/antibiotics11111632 (PMC9686812; doi:10.3390/antibiotics11111632)
Supplement: Supplementary file 1 [file antibiotics-11-01632-s001.zip › Supplementary Figure 1.pdf]

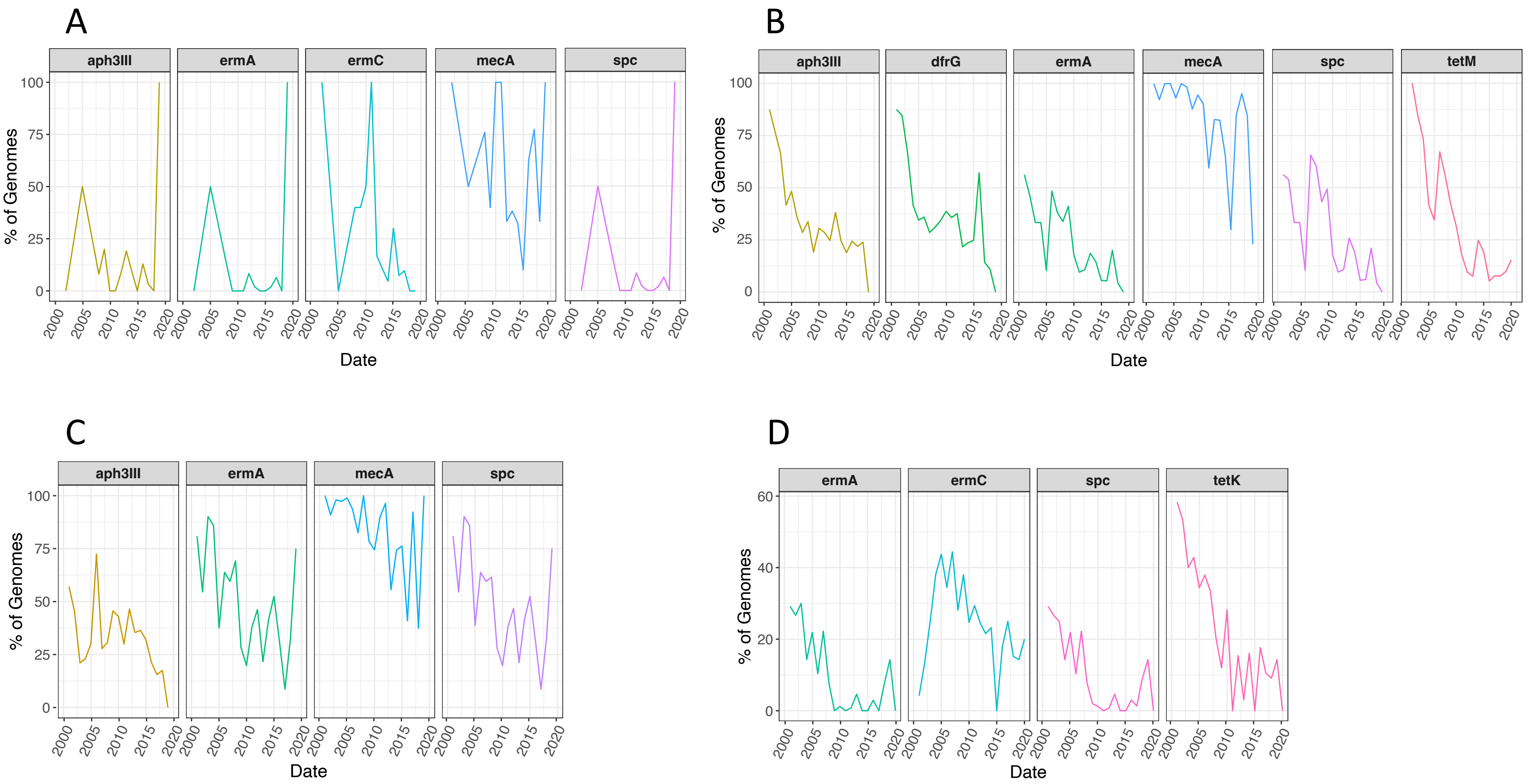

Supplemental Figure 1. Temporal changes on the occurrence of ARGs in the chromosomes of isolates from Africa (A), Asia (B), North/South America (C) and in the plasmidic contigs of Isolates from Asia (D). Only the ARGs described in the text are shown.
